# Impact of short basic emergency medicine training in introducing emergency medicine as a specialty in Sub-Saharan Africa: experience from Tanzania

**DOI:** 10.1186/s12245-018-0218-3

**Published:** 2019-01-11

**Authors:** Peter S. Mabula, Hendry R. Sawe, Victor Mwafongo, Juma A. Mfinanga, Michael S. Runyon, Brittany L. Murray

**Affiliations:** 1 Emergency Medicine Department, Muhimbili University of Health and Allied Science, Dar es Salaam, Tanzania; 2 Emergency Medicine Department, Muhimbili National Hospital, Dar es Salaam, Tanzania; 3 Department of Emergency Medicine, Carolinas Medical Center, Charlotte, NC USA; 4 Division of Pediatric Emergency Medicine, Emory University School of Medicine, Atlanta, GA USA

**Keywords:** Emergency medicine, Basic EM training in Tanzania, Tertiary hospitals

## Abstract

**Background:**

Emergency medicine (EM) is a new specialty in Tanzania. Little is known about how to introduce EM to health care providers (HCPs) in hospitals without EM. We determined the impact of a 2-day EM training program on the understanding, perception, and choice of EM as a career amongst HCPs at hospitals in Tanzania without EM.

**Methods:**

This was a pre- and post-training interventional study including randomly selected HCPs from four tertiary hospitals in Tanzania without EM. Understanding, perception, and desirability of EM as a career were assessed before and after a 2-day university-accredited basic EM short-course training given by EM physicians. A paper-based Likert scale (out of 5) questionnaire was used, which were analyzed by *T* tests, Mann-Whitney, and Kruskal-Wallis tests.

**Results:**

During the study period, 96 health care providers (100% capture) in the four tertiary hospitals participated in the study. The median age of participants was 34 years (IQR 28–43); 35 (36.0%) were males. Sixty (63%) were nurses, 26 (27%) doctors, and 3 (3%) were administrators. The four hospitals were equally represented. Median pre-training scores for all Likert questions were 3.49 (IQR 3.3–3.9); understanding 3.3 (IQR 3.0–3.7), perception 3.40 (IQR 3.1–3.7), and career decision-making 3.7 (IQR 3.3–4.0). Post-training scores improved with median scores of 4.6 (IQR 4.5–4.7) overall, 4.7 (IQR 4.0–4.7) for understanding, 4.6 (IQR 4.5–4.9) for perception, and 4.7 (IQR 4.3–4.8) for career decision-making (all *p* < 0.01).

**Conclusion:**

A 2-day training in basic EM care had a positive impact on understanding, perception, and career decisions regarding EM amongst Tanzania HCPs that work in hospitals without EM. Follow-up to assess long-term impact, and expansion of this program, is recommended to foster EM in countries where this is a new specialty.

## Introduction

In Tanzania, EM is a very new specialty [1]. In 2010, Tanzania established the first EM training program on the African continent outside of South Africa, at Muhimbili University of Health and Allied Sciences (MUHAS) in Dar es Salaam [1]. At the same time, a full capacity Emergency Medicine Department (EMD) was also established at Muhimbili National Hospital (MNH) [2]. The goal of the training program was to promote and spread sustainable emergency care across the country of Tanzania by training leaders in the field. This program has provided a unique opportunity for the country EM training and is expected to disseminate high-quality EM care to all Tanzanians. Since the establishment of this training program, graduates have been posted in different hospitals within Dar es Salaam where there have never been formal EM departments before. In recognition of the importance of EM, in 2014, MUHAS incorporated EM as a course in the undergraduate medical school curriculum.

Tanzania’s health care system is provided in a pyramidal system beginning with dispensaries at the lowest level, which refers patients to health centers, and then district hospitals, and regional and tertiary referral hospitals. There is a variable level of emergency care capacity with no EM specialists or formal full-capacity emergency departments (EDs) except for MNH which has a full capacity EMD [3]. Until the inception of emergency medicine, hospitals in Tanzania, like many low- and middle-income countries (LMICs) have handled emergencies through designated acute areas (commonly referred as casualty). These are outpatient departments (OPDs), usually minimally equipped and staffed with HCPs with no or little emergency care training. They largely channel patients to wards, theaters, and clinics and provide little or no emergency stabilization. As a result, EM graduates from Muhimbili practicing outside of MNH have encountered many challenges in their new working places. Graduates reported that their fellow HCPs had a poor perception of EM as a specialty and a poor understanding of what EM is and what it does. This was compounded by the hospital administration, which did not provide the appropriate equipment and supplies for EM care delivery.

One graduate, D. Yash, stated, “it has taken a long time to be recognized and be respected as EM specialists” (personal communication, July 3, 2015, MUHAS) [4].

In 2017, MUHAS will graduate EM physicians who have been tasked to lead the creation of EM departments and care at tertiary hospitals across Tanzania outside of Dar es Salaam city. These EM physicians are expected to face similar, and perhaps greater challenges, outside of Dar es Salaam, a city now familiar with EM. Similar to early years of EM specialty programs that are now well developed and established throughout the world [5, 6], the understanding, perception, and desirability of EM as a career amongst HCPs in the region poses a challenge to short- and long-term plans for developing and sustaining full-capacity EM departments at these hospitals.

We sought to determine the impact of an intervention to change the attitudes on understanding and perceptions of HCPs at referral hospitals in Tanzania where the graduates will soon establish EM departments. Finding interventions that might impact career choices of the HCPs in favor of EM will be very useful for the long-term sustainability and spread of this specialty within these tertiary hospitals and Tanzania at large. The data gathered from this study will be used to address the challenges of the current and subsequent groups of EM graduates and inform institutions within and beyond Tanzania that may have EM programs at various stages of development.

## Methods

### Study design

This was an interventional study evaluating attitudes and perceptions of EM as a specialty before and after a 2-day, basic EM training course. The study was conducted between June 2016 and March 2017. Pre- and post-training paper questionnaires with Likert scale variables.

### Study setting

**Fig. 1:**
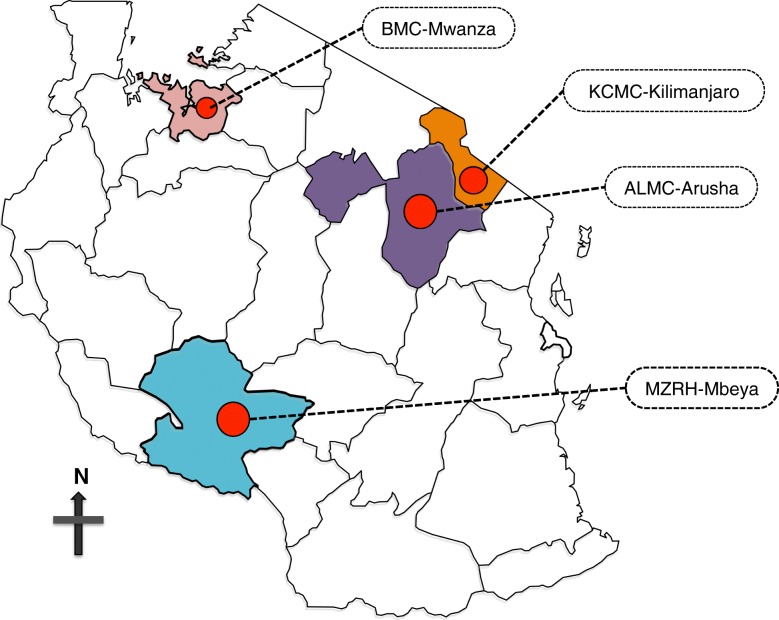
Map of Tanzania showing the locations of hospitals. The tertiary referrals are geopolitically located to serve different zones of Tanzania, to ensure wide populations of patients have access to tertiary care. Patients from each of the geopolitical zones are referred in line with a close proximity of their hospital to a respective zonal hospital. This figure was modified with permission from yourfreetemplates.com

The study was conducted in four tertiary referral hospitals in Tanzania where the 2017 EM graduates from MNH will be establishing new EM programs: Arusha Lutheran Medical Centre (ALMC), Kilimanjaro Christian Medical Centre (KCMC), Bugando Medical Centre (BMC), and Mbeya Zonal Referral Hospital (MZRH) (Fig. 1).

ALMC is a faith-based, private tertiary referral hospital located in the city of Arusha with a bed capacity of 125, mean while KCMC is a semi-public/private teaching hospital located in Kilimanjaro, Tanzania, with a bed capacity of 630 both located in Northern Tanzania. BMC is a faith-based, not-for-profit teaching hospital located in Mwanza city North-West of Tanzania with a bed capacity of 900, and MZRH is a public, zonal referral hospital run by the Ministry of Health of Tanzania, located in Mbeya city in the southern highlands of Tanzania with a bed capacity of 477. All of these hospitals have no formal EM departments; instead, they have OPDs which are run by doctors with no formal EM training except for ALMC where they have been receiving foreign emergency physicians.

### Participants

HCPs from each of four hospitals were eligible and selected at random by hospital administration. To ensure representation from each staff group, participants at each hospital were first clustered according to their job description (cadre) and then the total number of every cadre was divided by the total number of hospital staff to determine the percentage of staff represented by that cadre. The resulting percentage was multiplied by 25 to get the number of participants required for each cadre to participate in the study. Once these numbers were specified, the hospital administration was requested to randomly sample the cadre to obtain the proper number for the training. Foreign HCPs on temporary permit, other HCPs on temporary contracts, and those who did not complete the full course were excluded.

### Intervention

A 2-day Pediatric Emergency Care Training (PECT) short course was utilized as an intervention. The course was developed by MUHAS-EM in collaboration with MNH-EMD and the Emergency Medicine Association of Tanzania (EMAT) and is accredited by MUHAS. PECT content includes assessment, problem identification, and emergent management of a pediatric patient with medical and or trauma condition. PECT has a pre- and post-assessment exam, to help facilitators and trainees reflect on the level of knowledge before and after training, which was not part of this study since we had our own questionnaire. A post-training test is given, and participants with a score of 75% and above are awarded with MUHAS certificates of successful completion. The course was conducted by the principal investigator and other senior EM residents and EM specialist level physicians at each of the participating hospitals.

### Measurements

For the purposes of this study, we developed a questionnaire regarding understanding, perception, and attitudes toward choosing a career in EM. Questions regarding understanding addressed knowledge about what emergency medicine and what emergency physician do. Perception questions were those asking about the value, impact, and need for the specialty, while attitudes toward a career EM asked about whether this would be a desirable career for that person or others they were advising. Questions included Likert scale of 1 to 5, (1 for “*strongly disagree*” and 5 for “*strongly agree*”), variables (Appendix 1) and open-ended questions. Twenty emergency physicians and residents were selected as content experts to evaluate the clarity and relevance of each of the questions within the survey. The index of content validity (CVI) was then calculated for each [7, 8]; any question scoring 0.78 or greater was retained as it is, and those scoring < 0.78 were edited or deleted by expert consensus [7, 8]. The questionnaire was then piloted on HCPs at MNH who were not working in EMD. The questionnaire did not address course content, which was evaluated in a separate examination.

Participants in this study were consented and enrolled by the principal investigator. Each participant was assigned a study number so that questionnaire answers would be anonymous and pre- and post-scores per individual could be compared.

### Data analysis

The study data was transferred from the paper questionnaires into Microsoft Excel spreadsheet for Mac 2015 version 15.18 (Microsoft Corporation, Redmond, WA, USA) for analysis. Likert scores were summed, and then, an average for each category computed for the group as a whole, and for each individual hospital.

To find out whether the distribution was normal or skewed, the quantitative data was run into StatsDirect Version 3.0.171 and then simple descriptive statistics were used to report demographics and pre- and post-quantitative results using median (IQR) results were analyzed. Differences between pre- and post-training responses were analyzed with paired *T* tests for means, Mann-Whitney test for medians and Kruskal-Wallis test. *P* values are reported, and a value of alpha < 0.05 is considered statistically significant.

## Results

During the study period, 96 HCPs in the four tertiary hospitals were enrolled and completed the 2-day training as well as the pre- and post-training surveys. The median age of participants was 34 years (IQR 28–43); 35 (36.0%) were males. Most of the participants were diploma holders, and 60% of all participants were nurses. Forty-five percent had worked as health care providers for 5 years or less. All main hospital departments were represented according to the size of each hospital (Table 1).

**Table 1:** Demographic characteristics of participants

		Overall	ALMC	KCMC	BMC	MZRH
Total number of participants	*N* (%)	96 (100.0)	25 (26.0)	21 (22.0)	25 (26.0)	25 (26.0)
Age: median (IQR) years		34 (28.0–43.0)	37 (28.0–47.0)	31 (27.0–44.0)	34 (28.0–43.5)	35 (26.0–42.5)
Male	*n* (%)	35 (36.0)	7 (28.0)	4 (19.0)	12 (48.0)	12 (48.0)
Education level						
Certificate	*n* (%)	23 (24.0)	4 (16.0)	2 (10.0)	5 (20.0)	12 (48.0)
Diploma	*n* (%)	43 (45.0)	12 (48.0)	9 (43.0)	13 (52.0)	9 (36.0)
Degree	*n* (%)	30 (31.0)	9 (36.0)	10 (47.0)	7 (28.0)	4 (16.0)
Cadres						
Doctors	*n* (%)	26 (27.0)	9 (36.0)	6 (29.0)	6 (24.0)	5 (20.0)
Nurses	*n* (%)	60 (63.0)	15 (60.0)	15 (71.0)	13 (52.0)	17 (68.0)
Administrators	*n* (%)	3 (3.0)	0 (0.0)	0 (0.0)	2 (8.0)	1 (4.00)
Others	*n* (%)	7 (7.0)	1 (4.0)	0 (0.0)	4 (16.0)	2 (8.0)
Working experience (years)						
Less than 5	*n* (%)	44 (46.0)	9 (36.0)	10 (48.0)	14 (56.0)	11 (44.0)
Department						
Internal medicine	*n* (%)	20 (21.0)	3 (12.0)	2(10.0)	8 (32.0)	7 (28.0)
Obstetrics/gynecology	*n* (%)	4 (4.0)	1 (4.0)	2 (10.0)	1 (04.0)	0 (0.0)
Pediatrics	*n* (%)	11 (11.0)	1 (4.0)	7 (3.3)	0 (0.0)	3 (12.0)
Surgery	*n* (%)	20 (21.0)	8 (32.0)	1 (4.0)	5 (20.0)	6 (24.0)
Anesthesia	*n* (%)	2 (2.0)	2 (8.0)	0 (0.0)	0 (0.0)	0 (0.0)
Casualty/OPD	*n* (%)	13 (14.0)	2 (8.0)	3 (14.0)	3 (12.0)	5 (20.0)
ICU	*n* (%)	7 (7.0)	2 (8.0)	2 (10.0)	2 (8.0)	1 (4.0)
Administration	*n* (%)	19 (20.0)	6 (24.0)	4 (19.0)	6 (24.0)	3 (12.0)
EM awareness						
Presence of formal EM	*n* (%)	22 (23.0)	9 (36.0)	13 (62.0)	0 (0.0)	0 (0.0)
Worked in EM department	*n* (%)	14 (15.0)	7 (28.0)	7 (33.0)	0 (0.0)	0 (0.0)
Family member treated at ED	*n* (%)	40 (42.0)	9 (36.0)	11 (52.0)	12 (48.0)	8 (32.0)

HCPs working in casualty, ICU, and administration, those most likely to be involved in setting up EM in the hospitals, constituted 23% of the sample. Twenty-two (23%) of respondents stated their hospital had an emergency department; these were participants from ALMC and KCMC which they do not currently have EM departments, only casualty, and 15% stated that they had previously worked in an ED.

### Overall pre- and post-training median scores

Overall, post-training scores were significantly higher than those given before training. There was a significant improvement in scores for all domains at all sites, with the exception of ALMC where scores for perception improved, but did not reach significance (Table 2).

**Table 2:** Overall pre- and post-training median scores

Category	Pre-training overall median (IQR) *N* = 96	Post-training overall median (IQR) *N* = 96	*P value
Understanding	3.33 (3.00–3.66)	4.66 (4.00–4.66)	< 0.01
Perception	3.40 (3.14–3.71)	4.63 (4.50–4.87)	< 0.01
Career decisions	3.66 (3.33–4.00)	4.66 (4.33–4.83)	< 0.01

^*^Mann-Whitney test was used to calculate the *P* value

### Pre- and post-training scores on understanding

We looked separately at the understanding of health care providers across all hospitals of the study and found a statistically significant improvement in understanding after training in comparison to the baseline understanding levels; ALMC scores for understanding improved significantly but were lower post-course than the other sites (Table 3).

**Table 3:** Pre- and post-training differences on understanding by site

Hospital name		Understanding		
		Pre-training median (IQR)	Post-training median (IQR)	*P value
Overall	*N* = 96	3.33 (3.00–3.66)	4.66 (4.33–4.83)	< 0.01
ALMC	*N* = 25	3.66 (3.00–4.33)	4.33 (4.00–4.50)	< 0.04
KCMC	*N* = 21	3.66 (3.00–4.33)	4.83 (4.58–4.83)	< 0.01
BMC	*N* = 25	3.33 (3.00–3.33)	4.66 (4.50–5.83)	< 0.01
MZRH	*N* = 25	3.00 (3.00–3.66)	4.83 (4.50–4.83)	< 0.01

^*^Mann-Whitney test was used to calculate the *P* value

### Pre- and post-training scores on perception

When assessing perception, there was a statistically significant improvement post-training in all sites except for ALMC; this site began with a higher baseline score perception, and after training, there was a slight improvement, which was not statistically significant (Table 4).

**Table 4:** Pre- and post-training differences on perception by site

Hospital name		Perception		
		Pre-training median (IQR)	Post-training median (IQR)	*P value
Overall	*N* = 96	3.57 (3.14–4.28)	4.63 (4.50–4.87)	< 0.01
ALMC	*N* = 25	4.57 (4.14–4.85)	4.62 (4.37–5.00)	0.18
KCMC	*N* = 21	3.64 (3.14–4.28)	4.93 (4.81–5.00)	< 0.01
BMC	*N* = 25	3.42 (3.14–3.57)	4.62 (4.50–5.75)	< 0.01
MZRH	*N* = 25	3.28 (3.00–3.87)	4.50 (4.50–4.75)	< 0.01

^*^Mann-Whitney test was used to calculate the *P* value

### Pre- and post-training scores on career decision-making

When assessing the outcome on career decision-making, results showed significant improvement after training at each hospital (Table 5).

**Table 5:** Pre- and post-training differences on career decision-making by site

Hospital name		Career decision-making		
		Pre-training median (IQR)	Post-training median (IQR)	*P value
Overall	*N* = 96	3.66 (3.33–4.00)	4.66 (4.33–4.83)	< 0.01
ALMC	*N* = 25	4.00 (3.66–4.33)	4.33 (4.00–4.50)	< 0.01
KCMC	*N* = 21	3.50 (3.25–3.91)	4.83 (4.58–5.00)	< 0.01
BMC	*N* = 25	3.66 (3.50–3.83)	4.66 (4.50–4.83)	< 0.01
MZRH	*N* = 25	3.33 (3.16–3.83)	4.83 (4.50–4.83)	< 0.01

^*^Mann-Whitney test was used to calculate the *P* value

## Discussion

We found that a 2-day training program in basic EM skills, administered by emergency physicians, had a significant positive impact on the understanding, perception, and desirability of EM as a career. In addition to HCPs, there was representation from administrators who are a key component in the establishment of EM [3, 5].

Nearly half of the participants had work experience of 5 years or less suggesting they had recently graduated from their respective schools. Over 20% of our study participants––all from ALMC and KCMC—reported that their hospitals had formal EM departments; furthermore, 15% reported to have worked in a formal ED prior to this study. This was an interesting observation highlighting a potential gap in understanding of EM and its specialty because none of these participants had worked at EMD-MNH—the only full-capacity ED at the time of study—or outside Tanzania, in countries where full-capacity EDs exist. The specialty of EM in Tanzania is very new, and the first undergraduate training program within medical universities in Tanzania started in 2015 [1]; hence, none of these participants would have been exposed to the training or rotation. We believe exposure is necessary to impact health care provider awareness, as prior study has shown that formal exposure through a short training is necessary for providers to be aware of the specific specialty [9].

In this study, we found that the baseline level outcome in understanding, perception, and career decision-making was moderate across all hospitals. This is an interesting finding, given that one would expect a moderate level of understanding, and perception to lead to a positively biased response on the belief of the need for EM physicians in Tanzania. We believe this is because EM is still in infancy stage in Tanzanian, and hence, most of providers might have heard about the practice of emergency medicine from different sources but not have had an opportunity to witness the impact of EM [2]. The first undergraduate training program within medical universities in Tanzania started in 2015 [2]; hence, none of these participants would have been exposed to the training or rotation. About 20% of our study participants—all from ALMC and KCMC—reported that their hospitals had formal EM departments, and 15% reported to have worked in a formal ED prior to this study. This finding further highlights the gap in understanding of the specialty of EM because none of these participants had worked at EMD-MNH, the only full-capacity ED at the time of study, or outside Tanzania, in countries where full capacity EDs exist.

As further evidence of the need for this exposure, despite the overall moderate level in understanding, perception, and career decision-making, ALMC had the highest score in perception of EM as a specialty. This hospital has had a long-time affiliation with a USA ED that sends EM faculty yearly to mentor and support the clinical capacity within the urgent care [10]. The rest of hospitals had no similar arrangement.

Our training was mainly focused on exposing health care providers to the role of EM in caring for acutely ill children presenting to ED. Similar to published literature on different settings, we observed a significant improvement after the course in understanding, perception, and career decision-making from all participants at KCMC, BMC, and MZRH. However, ALMC observed non-significant change on perception, possibly because participants were already familiar with EM. Scores for understanding improved significantly here, but they were lower post-test than other sites. ALMC was the first site to have the course: it is possible that teaching improved in subsequent courses as instructors became aware of and could anticipate learners’ questions.

This study highlighted that a short basic EM training is a good way to impact HCPs in their understanding, perception, and career decision-making in areas where there is no established EM. It has been shown in other studies that a deep understanding of a specialty is essential for someone to choose a particular future career specialty [9].

### Limitations

This study was conducted in only four tertiary hospitals; hence, the results may not necessarily be generalizable to other HCPs in regional and district hospitals across Tanzania. The long-term impact of this training was not assessed by this study. Participants were chosen by the hospital administration after clustering techniques; hospital administration was instructed to select HCPs randomly, but this could have been a source of selection bias, since the selection at this stage was out of direct control. We did not assess the success of the course in improving knowledge and skills in emergency care; the course is one that has been previously given to different health care facilities and shown to be successful in imparting that knowledge.

## Conclusions

A short basic EM Training is feasible and acceptable and has an immediate positive impact on understanding, perception, and career decision-making amongst HCPs of tertiary hospitals in Tanzania EM as a specialty. Although the long-term impact needs to be assessed, a short basic EM training can introduce and improve the image of EM at hospitals who do not have this specialty.

## Appendix

**Table 6:** The following Likert scale questions with their pre- and post-median scores

Understanding	Pre	Post
1. Do emergency medicine physicians help prevent illnesses or injuries?	3.76	4.50
2. Do emergency physicians work along emergency-trained nurses?	3.28	4.27
3. Do emergency medicine physicians prevent secondary injury to patients?	3.29	4.68
4. Does establishment of dedicated emergency care services reduce morbidity?	3.61	4.48
5. Does establishment of dedicated emergency care services reduce mortality?	3.40	4.45
Perception		
6. Do you think you need emergency medicine at your hospital?	3.41	4.37
7. Do you think the introduction of emergency medicine care in Tanzania will help save more lives than before?	4.01	4.78
8. Do you think emergency medicine physicians are respected by other physicians?	3.85	4.71
9. Do you think emergency medicine physicians and their practice in Tanzania will change the way patients with emergency conditions are been handled?	3.60	4.61
10. Do you think district and region hospitals should have emergency medicine physicians?	3.58	4.66
Career decision-making		
11. Do you think nursing/medical students should rotate in emergency medicine?	3.95	4.63
12. How often do emergency medicine physicians get called from home?	3.79	4.73
13. Do you think emergency medicine offers a good future financial reward?	3.73	4.55
14. Do you think emergency medicine physicians are satisfied by career choice?	3.28	4.48
15. Would you advise someone to pursue emergency medicine as a specialty?	3.61	4.45
16. Do professionals in emergency medicine in Tanzania have career opportunities?	3.52	4.67
17. Would you advise a medical student to become an emergency medicine physician?	3.60	4.68

## Abbreviations

ALMC: Arusha Lutheran Medical Centre
AMO: Assistant Medical Officer
BMC: Bugando Medical Centre
ED: Emergency department
EGD: Esophageal gastroduodenoscopy
EM: Emergency medicine
EMAT: Emergency Medicine Association of Tanzania
EMD: Emergency Medicine Department
EMS: Emergency Medical Services
HCP: Health care providers
KCMC: Kilimanjaro Christian Medical Centre
MUHAS: Muhimbili University of Health and Allied Sciences
MZRH: Mbeya Zonal Referral Hospital
PECT: Pediatrics Emergency Care Training
